# A Computational Approach for Understanding the Interactions between Graphene Oxide and Nucleoside Diphosphate Kinase with Implications for Heart Failure

**DOI:** 10.3390/nano8020057

**Published:** 2018-01-23

**Authors:** Anushka Ray, Isaac Macwan, Shrishti Singh, Sushila Silwal, Prabir Patra

**Affiliations:** 1 Nashua High School South, Nashua, NH 03062, USA; anushkaray101@gmail.com; 2 Department of Biomedical Engineering, University of Bridgeport, Bridgeport, CT 06604, USA; shrsingh@my.bridgeport.edu (S.S.); ssilwal@my.bridgeport.edu (S.S.); ppatra@bridgeport.edu (P.P.)

**Keywords:** nucleoside diphosphate kinase, graphene oxide, molecular dynamics, heart failure

## Abstract

During a heart failure, an increased content and activity of nucleoside diphosphate kinase (NDPK) in the sarcolemmal membrane is responsible for suppressing the formation of the second messenger cyclic adenosine monophosphate (cAMP)—a key component required for calcium ion homeostasis for the proper systolic and diastolic functions. Typically, this increased NDPK content lets the surplus NDPK react with a mutated G protein in the beta-adrenergic signal transduction pathway, thereby inhibiting cAMP synthesis. Thus, it is thus that inhibition of NDPK may cause a substantial increase in adenylate cyclase activity, which in turn may be a potential therapy for end-stage heart failure patients. However, there is little information available about the molecular events at the interface of NDPK and any prospective molecule that may potentially influence its reactive site (His118). Here we report a novel computational approach for understanding the interactions between graphene oxide (GO) and NDPK. Using molecular dynamics, it is found that GO interacts favorably with the His118 residue of NDPK to potentially prevent its binding with adenosine triphosphate (ATP), which otherwise would trigger the phosphorylation of the mutated G protein. Therefore, this will result in an increase in cAMP levels during heart failure.

## 1. Introduction

The American Heart Association reports that the predicted population diagnosed with heart failure will rise to 46% by 2030 [1]. The most prevalent feature of a failing heart is when the systolic and/or diastolic functions are curtailed due to an imbalance in intracellular calcium ion (Ca^2+^) homeostasis in cardiomyocytes. In a non-failing heart, a cardiac contraction is initiated when an inflow of Ca^2+^ is released through L-type Ca^2+^ channels. This external influx prompts a large amount of Ca^2+^ from the internal stores in the cardiac sarcoplasmic reticulum to be released into the sarcoplasm. Subsequently, the calcium ions that are pumped out interact with the contractile proteins that use ATP to contract the muscle fiber, a process known as excitation-contraction coupling, where an electrical stimulus is converted into a mechanical response. An essential component for the maintenance of intracellular Ca^2+^ homeostasis is the diffusible intracellular second messenger cyclic adenosine monophosphate (cAMP): a product of the beta-adrenergic pathway that activates protein kinase A (PKA), which is, in turn, an enzyme dependent on cAMP activity and concentration [2]. PKA activates the Ca^2+^ channels and many other components of the cardiac excitation-contraction process [3].

A key player in the beta-adrenergic pathway needed for cAMP production is nucleoside diphosphate kinase (NDPK): a ubiquitous enzyme that plays a plethora of roles in the body such as phosphorylation, regulating various types of membrane enclosures that absorb dying cells during development, and regulating metastasis through its ability to take in bacteria or micronutrients [4,5]. It is now known that NDPK is found in the hearts of many species and is a key player in heart failure [6]. 

**Figure 1:**
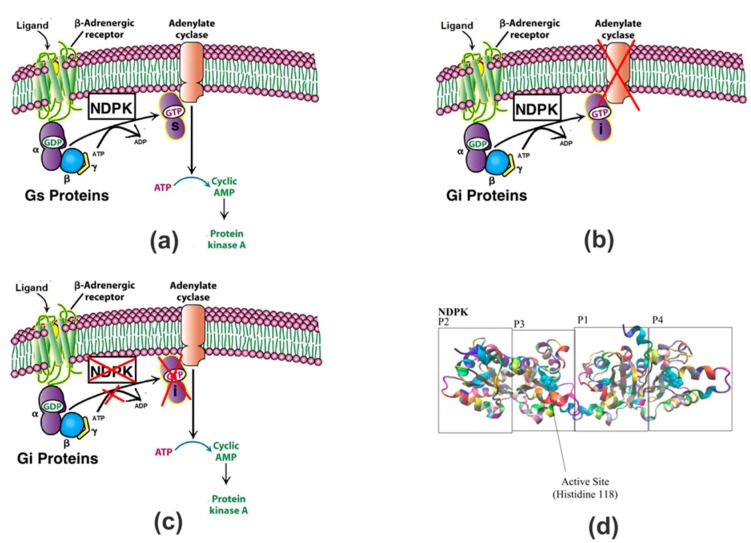
(**a**) The role of nucleoside diphosphate kinase (NDPK) in a non-failing heart. Typically, G_βγ_ binds with G_α_ and this guanosine diphosphate (GDP(s)) as a whole is further converted to Guanosine -5′-Triphosphate (GTP(s)) with the help of NDPK that triggers the adenylate cyclase in a dependent pathway to synthesize the cAMP; (**b**) Role of NDPK in a failing heart. During heart failure, the same NDPK inhibits the adenylate cyclase activity due to its increased interaction with cAMP inhibitory membrane proteins in the form of GDP(i), which is the result of the binding between G_βγ_ and G_α_(i), and the eventual conversion of GDP(i) to GTP(i); (**c**) Potential effects that may result due to graphene oxide inhibiting NDPK. The inhibition of NDPK by graphene oxide during heart failure can prevent the suppression of cAMP formation through the dependent pathway. Adenylate cyclase activity is resumed because the NDPK molecules reacting with G_i_ proteins are inhibited and the faulty GTP(i) is not formed anymore. The NDPK independent pathways continue to produce cAMP without the suppression that was occurring due to NDPK molecules reacting with the G_i_ proteins; (**d**) The structure of NDPK and the active sites on each monomer. Monomers are defined by labels: P1, P2, P3, P4 in Visual Molecular Dynamics (VMD)—a molecular graphics program. (Figures a–c are modified from Source: *Biochemistry, Sixth Edition* © 2007 W.H Freeman and Company).

NDPK is responsible for transferring a phosphate group from ATP to GDP, and this process is illustrated in Figure 1a. More specifically, the beta-gamma dimer binds to NDPK and receives a phosphate from NDPK’s active residue His118, onto its own residue His-266 [7]. Figure 1d shows the structure of NDPK and the location of the histidine residue.

Adrenergic receptors are comprised of G proteins that contain alpha, beta, and gamma subunits [8]. G proteins are heterotrimeric proteins containing 3 subunits and having their own family of proteins [9]. The alpha subunit attached to the beta-adrenergic receptor is called the G_s_ Alpha subunit. This subunit is responsible for stimulating the cAMP-dependent pathway by binding to the NDPK and thereby contributing to the activation of adenylate cyclase [10]. During end-stage heart failure, however, there is a 300% increase in NDPK content in the cardiac sarcolemmal membrane [7]. Normally, this overexpression of NDPK would cause an abundance of cAMP molecules. However, there is also a drastic increase in the levels of catecholamine, which affects the signaling of G proteins [11]. Therefore, this increase in the NDPK, in addition to an increase in G_i_ alpha subunits, results in the NDPK molecules reacting predominantly with the G_i_ (GDP(i)) inhibitory proteins in a dependent pathway. This is then responsible for inhibiting cAMP [12,13]. A direct consequence of this pathway is expressed in Figure 1b, indicating that the Guanosine-5′-Triphosphate (GTP) formed through NDPK phosphorylation during heart failure is responsible for inhibiting adenylate cyclase activity and restricting the conversion of ATP to cAMP to PKA. However, from past research, it has been shown experimentally that inhibition of NDPK in failing hearts causes a substantial increase in adenylate cyclase activity [14]. Furthermore, G protein pathways can be receptor-dependent or receptor-independent. The pathways indicated by Figure 1a,b represent receptor-dependent pathways because the cascade of reactions take place only when a ligand binds onto the receptor. However, receptor-independent pathways are also able to generate cAMP with only guanosine diphosphate (GDP) and adenosine triphosphate (ATP). One such study showed that when uracil diphosphate (UDP) was used to inhibit transphosphorylation in a healthy heart, stimulation of cardiac adenylate cyclase by GTP increased. The result was no change in the adenylate cyclase activity, indicating that such an independent pathway doesn’t involve NDPK. Figure 1c shows that if NDPK activity is restricted during heart failure, the inhibitory effects of NDPK can be hindered, and the receptor-independent pathway can continue to produce cAMP [15]. Research has shown that inhibiting NDPK results in favorable outcomes [16]. In order to test a potential solution, whereby blocking the active NDPK site may be beneficial in case of heart failure, this work analyzed the interactions between an oxidized carbon allotrope, graphene oxide (GO) and NDPK owing to interests in GO in biotechnology and biomedical fields. Nanomaterials like carbon nanotubes have been shown not inducing cell death. Therefore, they have relatively low toxicity [17]. More specifically, GO has been shown to be potentially applied in gene and drug delivery, cancer therapeutics, cellular imaging, as an antibacterial agent and bio-sensing [18,19,20,21,22]. Furthermore, GO is an ideal enzymatic substrate because it is saturated with oxygen-containing groups, allowing it to interact with enzymes without modification of the surface [23]. Additionally, GO is also a model substrate because it is biocompatible, has a large surface area, hydrophilic nature and has excellent colloidal stability in water [24,25]. This is the first report of the computational understanding of the interactions of NDPK in general and the phosphorylation site (histidine118) on the NDPK in particular with GO substrate. An all atom molecular dynamics simulation approach is utilized to quantify the interactive events at the interface of GO and NDPK towards potential applications for new therapies in case of heart failure.

## 2. Results and Discussion

**Figure 2:**
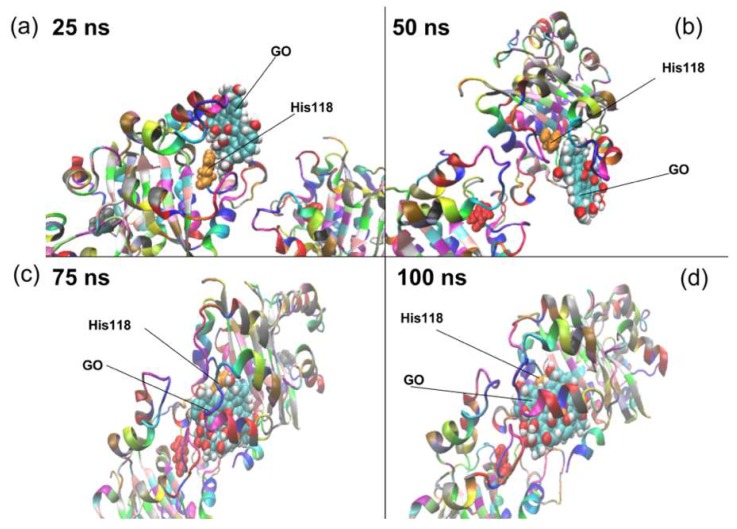
The position of the graphene oxide at (**a**) 25 ns; (**b**) 50 ns; (**c**) 75 ns and (**d**) 100 ns intervals are shown relative to the positions of the His118 residue and the NDPK enzyme. It can be seen that by 50 ns, graphene oxide starts interacting with the His118 residue and by 100 ns, graphene oxide completely blocks His118 residue’s interaction with the environment. NDPK is represented as a cartoon with a coloring style based on individual residues and His118 and graphene oxide are shown in the CPK representation showing the oxygen and hydrogen functionalities in red and white with carbon in cyan respectively.

To study the molecular events taking place at the interface of GO and NDPK, the simulated trajectory from the all-atom simulations of 100 ns was utilized. Through the molecular graphics program, VMD, the behavior of NDPK atoms with respect to GO was studied as shown in Figure 2 at different times during the 100 ns period. It can be seen from Figure 2 that the GO flake closest to the NDPK segments P1 and P3 directly interacted with the reactive site of NDPK (His118) after scanning the surface of NDPK for ~60 ns to locate a favorable conformation of the enzyme. The rest of the GO flakes showed attraction to the NDPK as well, but were not able to bind to the enzyme like GO5. The implications of these interactive events directly relate to the phosphorylation ability of NDPK indicating that NDPK can be constrained from binding to ATP for the exchange.

**Figure 3:**
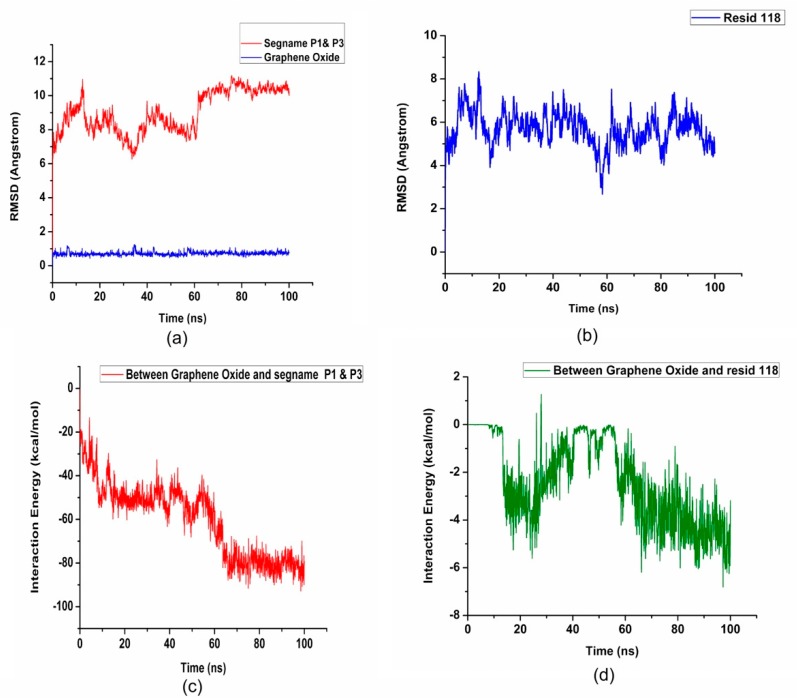
(**a**,**b**) show the Root Mean Square Deviation (RMSD) of the two middle NDPK monomers, graphene oxide (GO) flakes and the histidine residue 118 throughout the 100 ns simulation. Segment names P1 and P3 refer to the two middle monomers of the NDPK enzyme defined earlier in Figure 1d; (**c**) Interaction Energy between GO and the two middle NDPK monomer segments P1 and P3; (**d**) Interaction energy between GO and His118 residue.

In order to investigate further and explore the energies and biophysical phenomena accompanying such an interaction, VMD’s Analyses Plugins were utilized for studying the root mean square deviation (RMSD), non-binding energies such as Van der Waals and electrostatic (summation of both being the interaction energy), conformational energies for bonds, angles and dihedrals and the effect of such an interaction on the secondary structure of NDPK. RMSD is the deviation from the initial position of each component in the simulation. Figure 3a reveals that at ~60 ns from the beginning of the simulation run, NDPK stabilizes to a new conformation in the presence of GO. It is to be noted that the stability of both GO and NDPK at the threshold of binding is ~1 Å, which can be treated as a very stable and sustained complex formation. Interestingly, the RMSD of His118 also showed a stable behavior within 2 Å of the GO surface (Figure 3b).

Interaction energy is the summation of electrostatic attractions and the van der Waals forces between molecules, which is an atom-to-atom interaction and its integration over the entire adsorbed surface. Segments P1 and P3 were also analyzed for the total interaction energy and as can be seen from Figure 3c, maximum sustained interaction energy levels of ~85 kcal/mol between GO and NDPK were noticed after the 60 ns threshold that involved the 37 residues (Table 1) bound onto the surface of GO. Based on the number of residues bound, on an average the interaction energy per residue should be ~2.3 kcal/mol. However, looking at Figure 3d, it is found that His118 alone binds with three times this value (~6 kcal/mol by the end of the 100 ns run) clearly demonstrating the affinity of His118 to GO and hence gives a crucial insight into a potential therapy towards sustained cAMP synthesis in case of heart failure. Both the Root Mean Square Fluctuation (RMSF) and secondary structure analysis verify this affinity of His118 to the GO (Supplementary Figures S2 and S3). It is found that the RMSF of the His118 in the absence of GO (Figure S2A) is ~0.3 Å whereas in the presence of GO (Figure S2B), it varies from 0.445 to 3.134 Å indicating the molecular rearrangement in this key residue to find a favorable adsorption orientation on the surface of GO. Similarly, comparing the secondary structure of NDPK in the presence of GO (Figure S1) and in the absence of GO (Figure S3), it can be seen that the key residue His118 doesn’t lose its vital conformation as a part of the extended beta structure while interacting with the GO. This analysis based on the RMSD and the interaction energy calculations determined both strength and stability of the attractive forces between the enzyme and GO. It is to be noted that only the residues within 5 Å from the GO surface are considered to be directly influencing the process to be realistic in terms of the dominating short-range forces and most of these residues have a hydrophobic side chain.

**Figure 4:**
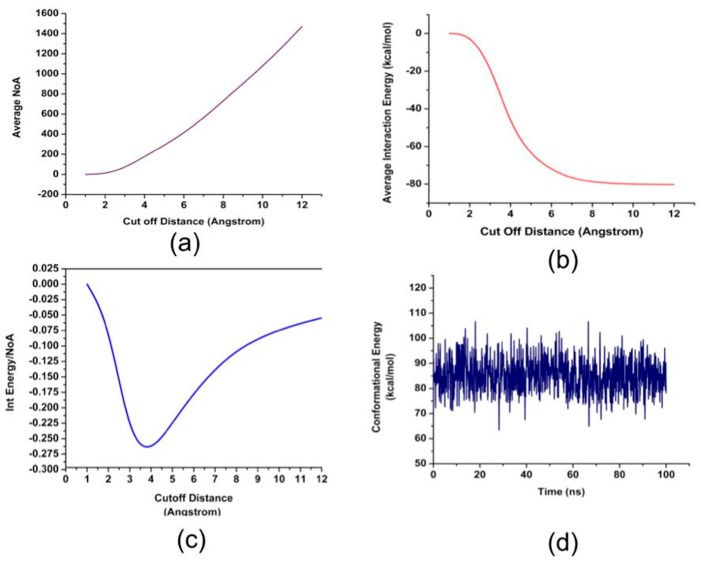
(**a**) The average number of atoms of the enzyme that are located from the respective cutoff distances from the graphene oxide flake; (**b**) The interaction energy between the enzyme and the graphene oxide as a function of the cutoff distance; (**c**) The interaction energy divided by the number of atoms as a function of the cut-off distance; (**d**) The conformational energies throughout the 100 ns.

The interaction energy, which is largely due to the Van der Waals forces in the present case is directly related to the surface area of the enzyme interacting with GO. Thus, in order to understand the role of the 18 hydrophobic residues with 5 Å of GO, relative cut-off distances between the center of mass of GO and the center of mass of the hydrophobic residues, and the associated interaction energies were analyzed (Figure 4). Interestingly, it was discovered that the optimum cut-off distance for maximum interaction energy per atom is ~3.75 Å. As can be seen from Figure 4a,b, the number of atoms and interaction energy because of these atoms were calculated relative to the cut-off distances from 1 to 12 Å. Figure 4c shows the optimum cut-off distance by determining the ratio of the number of atoms to the associated interaction energies. Furthermore, minor conformation changes to accommodate the interactions is visible from Figure 4d, which shows the total conformation energy shifting its average value ~60 ns in the simulation run coinciding with the threshold of complex formation between GO and NDPK as NDPK finds a favorable location on the surface of GO. These minor conformational changes are expected and the retaining of the conformational energy to a new value also confirms the stable conformation of the enzyme.

**Figure 5:**
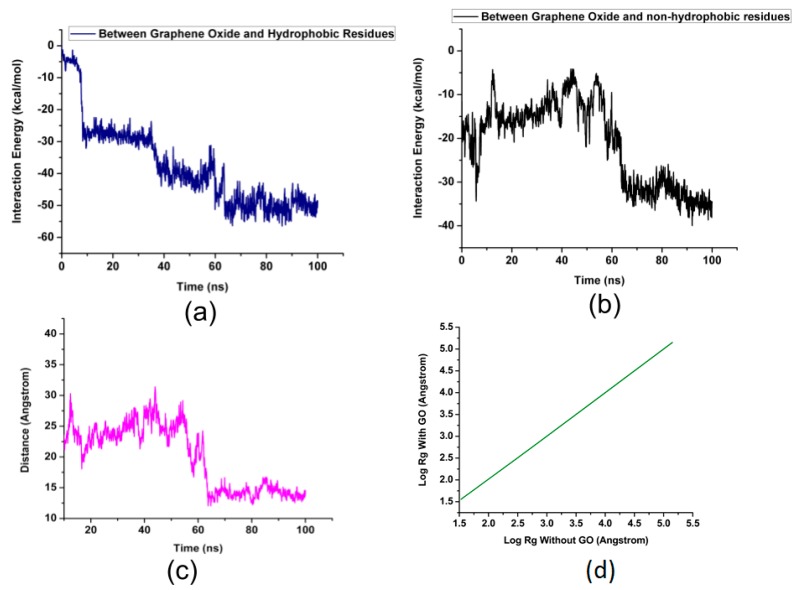
(**a**) The interaction energy between graphene oxide and the hydrophobic residues listed in Table 3; (**b**) The interaction energy between graphene oxide and the non-hydrophobic residues listed in Table 3; (**c**) The distance in angstroms between the center of mass of the graphene oxide and the primary residue; (**d**) Radius of gyration of NDPK in the presence and absence of GO.

In order to compare the energies of binding between the 18 hydrophobic residues and the rest of the residues within 5 Å from the surface of GO, two separate interaction energy profiles are generated. Figure 5a,b shows the difference in the interaction energies due to the 17 hydrophobic residues and rest of the 37 bound residues of NDPK. It can be seen that the interaction energy between GO and the hydrophobic residues reach levels of upto −60 kcal/mol and that the interaction energy between GO and the non-hydrophobic residues reach levels of ~−40 kcal/mol. This shows that GO has greater affinity for the hydrophobic residues. 

Figure 5c shows the change in the distance between the center of mass of GO and the center of mass of His118 indicating the affinity of His118 to GO, where His118 moved from a distance of ~25 Å down to less than 5 Å in about 60 ns. This rapid reduction in the distance between the two also is indicative of strong Van der Waals attractions and a crucial role that the 17 hydrophobic residues played in binding with GO.

The radius of gyration is a measure of the compactness of a protein. With the introduction of graphene-oxide, the radius of gyration remains the same. Figure 5d is a log-log plot of R_g_ in the presence and absence of GO. It signifies that NDPK has not lost its complete conformation with the introduction of GO. However, the strong binding of GO with NDPK makes GO an inhibitor of the enzyme, without compromising its 3D arrangement owing to a little difference between the R_g_ values of NDPK in the presence and absence of GO.

Table 2 displays the Solvent Accessible Surface Area (SASA) for regions in the enzyme in the presence and absence of GO. In its native state, in the absence of GO, 286 nm^2^ of NDPK’s surface area is accessible to water. But, as GO is introduced near its active site, the amino-acids rearrange themselves resulting in a higher exposure of the protein to water as indicated by an increased SASA value of ~329 nm^2^. In the absence of GO, 45.24% of the NDPK’s hydrophobic residues are exposed to the solvent (water) but as GO is lodged in the active site of the enzyme, the hydrophobic residues rearrange themselves to interact strongly with GO as 24.31% of those residues are capable of interacting with water. This is a 20.9% decrease from the former value. The hydrophobic regions of NDPK are bound to GO, which is a good indicator of the inhibitory activity of GO as conserved domains (hydrophobic in nature) are attributed to the primary function of most proteins. Table 2 also displays SASA for the hydrophobic and hydrophilic regions of the individual segments in the parenthesis next to the values for the whole segments.

**Figure 6:**
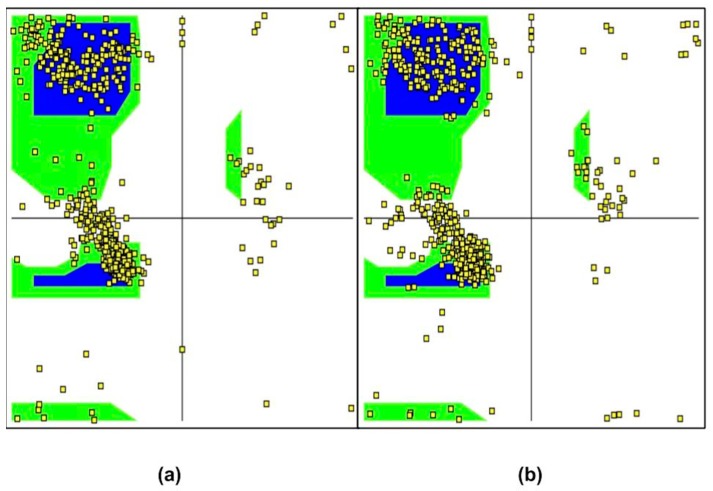
Ramachandran Plot in the (**a**) absence and (**b**) presence of GO. Blue and green regions indicate the favorable and allowed regions of conformation on the NDPK enzyme. Little yellow squares indicate the individual amino acid residues. There are no drastic changes observed in the presence and absence of GO indicating that NDPK doesn’t lose its function while strongly interacting with GO.

Figure 6a is the Ramachandran plot in the absence of GO. The blue and green regions on the plot indicate the favorable and allowed regions of conformation. In their native state, majority of the amino-acid residues (indicated by the yellow squares) in NDPK have permissible Φ and Ψ values in the favored and allowed regions when GO is absent. When GO is present with NDPK, as shown in Figure 6b, there are no drastic differences observed in the state of NDPK indicating that the protein does not lose its dominant alpha-helix structure but rearranges itself to interact strongly with GO at its active site.

While this study is important from the point of view of therapeutics in case of failing hearts, it is equally important to regulate the binding of GO with His118, thereby making sure that the secondary structure of the enzyme is intact and capable of performing its function as a phosphorylation intermediary in the event that GO is forced to unbind the reactive site. This led to the analysis of the secondary structure of NDPK post binding (Figure S1) and it is found that although GO is able to temporarily inhibit the function of NDPK, it does not damage the enzyme permanently. Together these results indicate that GO favorably interacted with NDPK and potentially blocked the phosphorylation site of NDPK.

## 3. Materials and Methods

The interactions between NDPK and GO were simulated using nanoscale molecular dynamics (NAMD): a molecular dynamics code designed to simulate large biomolecular systems [26]. The results from the simulation were analyzed using Visual Molecular Dynamics (VMD, University of Illinois–Urbana, Urbana, IL, USA): a molecular visualization program that is used for displaying, animating, and analyzing the systems that are simulated using NAMD. NAMD [27] and VMD [28] were used together to simulate and analyze the interactions between NDPK and GO. The necessary structural file for the NDPK was obtained from the pdb databank (2ZUA). The structural file for GO was created using molefacture plugin in VMD from the graphene sheet generated using an inbuilt graphene sheet builder plugin, which was then used to create six GO flakes (size 15.45 Å × 11.68 Å). The chemical structure of the flakes used is C_10_O_1_(OH)_1_(COOH)_0.5_, also known as OGO [29]. The pdb file of this structure was edited for the incorporation of the six GO flakes and their orientation through the x,y,z co-ordinates around the NDPK molecule in the pdb file was modified using a TCL script in the VMD. The flakes were placed so they were approximately equidistant from the active sites, namely the His118 residue. Their respective distances from the four His118 active sites on the enzyme are given in Table 3.

All-atom simulations were carried out between the six GO flakes and NDPK (~120,000 atoms) for a time period of 100 ns. All simulations used CHARMM (Chemistry at HARvard Macromolecular Mechanics) force field [30] with Transferable Intermolecular Potential with 3 Points (TIP3P) [31] water model and neutralizing concentration of NaCl to effectively polarize the water molecules. The Extreme Science and Engineering Discovery Environment (XSEDE) [32] supercomputing facility provided access to 54 Central Processing Units (CPU) pairs with 28 cores per CPU (1512 cores) supercomputer cluster on which simulations were carried out. The temperature was set to 300 K and maintained by Langevin Thermostat, the pressure was set to 1 atm. Through Nose-Hoover Langevin-piston barostat with a period of 100 ps and a decay rate of 50 ps. An integrated time-step of 2 fs was maintained for all simulations. A cut-off of 10–12 Å was used for short-range forces which particle mesh Ewald algorithm was used for calculating long-range forces. A 5000—step energy minimization was performed first on the entire system (GO, NDPK, water and ions) to reach a minimum for the potential energy followed by an equilibration of 500,000 steps (1 ns). Root Mean Square Deviation (RMSD) and NAMD Energy extensions were used from VMD to determine the interaction energies between the NDPK and the GO flakes. Additionally, TimeLine tool from VMD is utilized to analyze the secondary structure of the NDPK while it interacts with the GO flakes. Interaction energies, optimum cut-off distances from the surface of GO flakes and relative distance between the GO flakes and NDPK were quantified using TCL scripts.

## 4. Conclusions

In conclusion, this study analyzed the events occurring at the interface of GO and NDPK from the viewpoint of energetically favorable residues through molecular dynamics simulations. It is found that owing to the interactions between GO and the potential phosphorylation site through the residue histidine (118) on NDPK, cAMP synthesis can be regulated via the controlled blocking of this residue. During heart failure, cAMP production is curtailed due to NDPK increasing the activity of cAMP inhibitory pathways. The presence of NDPK in non-failing hearts increase the activity of cAMP stimulatory pathways. Therefore, the inhibition of NDPK activity using GO is temporary. This study can be extended in a multitude of ways through experiments and a more complex interactive analysis using only the major isoforms of NDPK (NDPK-B and NDPK-C) that are responsible for the remodeling of the heart in the case of heart failure. Furthermore, this study can also be applied to project interactive events of NDPK in general for other cellular processes and their regulation. For example, because NDPK plays a role in cell proliferation, GO can potentially be used to inhibit the undesired proliferation. Also, GO can be removed from the enzyme by inserting a stronger substrate that can take the place of GO by creating a competitive environment. Since the simulation showed that the residues of NDPK retained its secondary structure after interaction with GO, a “switch” can be established where NDPK can be disabled and enabled based on the circumstance. It is anticipated that inhibiting NDPK during heart failure will prevent cAMP production from being suppressed, which will help maintain cardiomyocyte Ca^2+^ homeostasis for proper cardiac contraction for a sustained periods of time, which can be beneficial for developing new therapies.

## Figures and Tables

**Table 1:** Residues within 5 Angstrom of Graphene Oxide Surface.

Residue Name	Residue ID	Residue Type
Histidine	122, 118	Basic, positively charged
Aspartic Acid	57, 124, 148, 14, 121	Acidic, negatively charged
Glutamic Acid	123, 125	Acidic, negatively charged
Tryptophan	144, 151	Aromatic, hydrophobic
Leucine	152, 64, 112	Aliphatic, hydrophobic
Tyrosine	153, 52	Aromatic, hydrophobic
Methionine	10	Sulfur-containing, hydrophobic
Lysine	12, 58	Basic, positively charged
Glycine	126, 63, 71, 113, 119	Aliphatic, hydrophobic
Proline	13, 59, 72	Aliphatic
Glutamine	42	Amidic, polar uncharged
Isoleucine	43, 68	Aliphatic, hydrophobic
Threonine	69	Hydrophilic, polar uncharged
Valine	73	Aliphatic, hydrophobic
Asparagine	115	Amidic, polar uncharged
Phenylalanine	60, 67	Aromatic, hydrophobic

**Table 2:** Solvent Accessible Surface Area (SASA).

Solvent Accessible Surface Area (SASA) (nm^2^)	Presence of GO	Absence of GO
Segment P1	96.71 (68, 109.61)	89.40 (22.54, 66.85)
Segment P2	97.80 (66.7, 111)	83.65 (22.83, 60.81)
Segment P3	104.70 (68.58, 116.52)	87.35 (21.76, 65.6)
Segment P4	97.70 (65.95, 110.9)	84.25 (21.52, 62.72)
NDPK (whole)	328.80	286.19
Hydrophobic region of NDPK	243.80	65.13
Hydrophilic region of NDPK	426.95	221.06

**Table 3:** Initial positioning of the GO flakes.

GO Flake	Active Site Location	Approximate Distance from Active Site (Angstroms)
GO1	P4	35.94
GO2	P4	31.61
GO3	P2	34.24
GO4	P2	28.85
GO5	P3	32.83
GO6	P1	29.98

## Supplementary Materials

The following are available online at http://www.mdpi.com/2079-4991/8/2/57/s1, Figure S1: Secondary structure timeline analysis computed by VMD’s timeline plug-in, Figure S2: Root Mean Square Fluctuation (RMSF) of the His118 residue from the four monomers, P1, P2, P3 and P4 in the absence (A) and presence (B) of graphene oxide; Figure S3: Secondary structure analysis of the NDPK enzyme in the absence of graphene oxide showing the His118 residue from the four monomers P1, P2, P3 and P4 undergoing conformational changes.

## References

[1] Benjamin E.J., Blaha M.J., Chiuve S.E., Cushman M., Das S.R., Deo R., de Ferranti S.D., Floyd J., Fornage M., Gillespie C. (2017). Heart Disease and Stroke Statistics—2017 Update: A Report From the American Heart Association. Circulation.

[2] Zaccolo M. (2009). cAMP signal transduction in the heart: Understanding spatial control for the development of novel therapeutic strategies. Br. J. Pharmacol..

[3] Luo M., Anderson M.E. (2013). Mechanisms of Altered Ca^2+^ Handling in Heart Failure. Circ. Res..

[4] Takács-Vellai K., Vellai T., Farkas Z., Mehta A. (2015). Nucleoside diphosphate kinases (NDPKs) in animal development. Cell. Mol. Life Sci..

[5] Randazzo P.A., Northup J.K., Kahn R.A. (1992). Regulatory GTP-binding proteins (ADP-ribosylation factor, Gt, and RAS) are not activated directly by nucleoside diphosphate kinase. J. Biol. Chem..

[6] Zhou Y.-Y., Artman M. (2001). Nucleoside diphosphate kinase: A new player in heart failure?. Cardiovasc. Res..

[7] Hippe H.-J., Luedde M., Lutz S., Koehler H., Eschenhagen T., Frey N., Katus H.A., Wieland T., Niroomand F. (2007). Regulation of Cardiac cAMP Synthesis and Contractility by Nucleoside Diphosphate Kinase B/G Protein βγ Dimer Complexes. Circ. Res..

[8] Kimura N. (1993). Role of Nucleoside Diphosphate Kinase in G-Protein Action.

[9] Pronin A.N., Gautam N. (1992). Interaction between G-protein 13 and y subunit types is selective (signal transduction/subunit families). Biochem. Commun. Melvin.

[10] Liu J., Erlichman B., Weinstein L.S. (2003). The Stimulatory G Protein α-Subunit Gsα Is Imprinted in Human Thyroid Glands: Implications for Thyroid Function in Pseudohypoparathyroidism Types 1A and 1B. J. Clin. Endocrinol. Metab..

[11] Abu-Taha I.H., Heijman J., Feng Y., Vettel C., Dobrev D., Wieland T. (2017). Regulation of heterotrimeric G-protein signaling by NDPK/NME proteins and caveolins: An update. Lab. Investig..

[12] Neumann J., Scholz H., Döring V., Schmitz W., Von Meyerinck L., Kalmárb P. (1988). Increase in myocardial G_i_-proteins in heart failure. Lancet.

[13] Abu-Taha I.H., Heijman J., Hippe H.-J., Wolf N.M., El-Armouche A., Nikolaev V.O., Schäfer M., Würtz C.M., Neef S., Voigt N. (2017). Nucleoside Diphosphate Kinase-C Suppresses cAMP Formation in Human Heart FailureClinical Perspective. Circulation.

[14] Lutz S., Mura R., Baltus D., Movsesian M., Kübler W., Niroomand F. (2001). Increased activity of membrane-associated nucleoside diphosphate kinase and inhibition of cAMP synthesis in failing human myocardium. Cardiovasc. Res..

[15] Niroomand F., Mura R., Jakobs K.H., Rauch B., Kübler W. (1997). Receptor-Independent Activation of Cardiac Adenylyl Cyclase by GDP and Membrane-Associated Nucleoside Diphosphate Kinase. A New Cardiotonic Mechanism?. J. Mol. Cell. Cardiol..

[16] Anciaux K., Van Dommelen K., Willems R., Roymans D., Slegers H. (1997). Inhibition of nucleoside diphosphate kinase (NDPK/nm23) by cAMP analogues. FEBS Lett..

[17] Aldinucci A., Turco A., Biagioli T., Toma F.M., Bani D., Guasti D., Manuelli C., Rizzetto L., Cavalieri D., Massacesi L. (2013). Carbon Nanotube Scaffolds Instruct Human Dendritic Cells: Modulating Immune Responses by Contacts at the Nanoscale. Nano Lett..

[18] Bianco A., Kostarelos K., Prato M. (2005). Applications of carbon nanotubes in drug delivery. Curr. Opin. Chem. Biol..

[19] Stiriba S.-E., Frey H., Haag R. (2002). Dendritic polymers in biomedical applications: From potential to clinical use in diagnostics and therapy. Angew. Chem. Int. Ed. Engl..

[20] Sun X., Liu Z., Welsher K., Robinson J.T., Goodwin A., Zaric S., Dai H. (2008). Nano-graphene oxide for cellular imaging and drug delivery. Nano Res..

[21] Li Q., Mahendra S., Lyon D.Y., Brunet L., Liga M.V., Li D., Alvarez P.J.J. (2008). Antimicrobial nanomaterials for water disinfection and microbial control: Potential applications and implications. Water Res..

[22] Wang J., Lin Y. (2008). Functionalized carbon nanotubes and nanofibers for biosensing applications. Trends Anal. Chem..

[23] Sun X., Feng Z., Hou T., Li Y. (2014). Mechanism of Graphene Oxide as an Enzyme Inhibitor from Molecular Dynamics Simulations. ACS Appl. Mater. Interfaces.

[24] Li S., Aphale A.N., Macwan I.G., Patra P.K., Gonzalez W.G., Miksovska J., Leblanc R.M. (2012). Graphene Oxide as a Quencher for Fluorescent Assay of Amino Acids, Peptides, and Proteins. ACS Appl. Mater. Interfaces.

[25] Kim J., Cote L.J., Kim F., Yuan W., Shull K.R., Huang J. (2010). Graphene Oxide Sheets at Interfaces. J. Am. Chem. Soc..

[26] Kumar S., Huang C., Zheng G., Bohm E., Bhatele A., Phillips J.C., Yu H., Kale L.V. (2008). Scalable Molecular Dynamics with NAMD on the IBM Blue Gene/L system. IBM J. Res. Dev..

[27] Phillips J.C., Braun R., Wang W., Gumbart J., Tajkhorshid E., Villa E., Chipot C., Skeel R.D., Kalé L., Schulten K. (2005). Scalable molecular dynamics with NAMD. J. Comput. Chem..

[28] Humphrey W., Dalke A., Schulten K. (1996). VMD: Visual molecular dynamics. J. Mol. Graph..

[29] Tang H., Liu D., Zhao Y., Yang X., Lu J., Cui F. (2015). Molecular Dynamics Study of the Aggregation Process of Graphene Oxide in Water. J. Phys. Chem. C.

[30] MacKerell A.D., Bashford D., Bellott M., Dunbrack R.L., Evanseck J.D., Field M.J., Fischer S., Gao J., Guo H., Ha S. (1998). All-Atom Empirical Potential for Molecular Modeling and Dynamics Studies of Proteins. J. Phys. Chem. B.

[31] Jorgensen W.L., Chandrasekhar J., Madura J.D., Impey R.W., Klein M.L. (1983). Comparison of simple potential functions for simulating liquid water. J. Chem. Phys..

[32] Towns J., Cockerill T., Dahan M., Foster I., Gaither K., Grimshaw A., Hazlewood V., Lathrop S., Lifka D., Peterson G.D. (2014). XSEDE: Accelerating Scientific Discovery. Comput. Sci. Eng..

