# Supplementary figures and images for: A Computational Approach for Understanding the Interactions between Graphene Oxide and Nucleoside Diphosphate Kinase with Implications for Heart Failure
Source: Nanomaterials (Basel). 2018 Jan 23;8(2):57. doi: 10.3390/nano8020057 (PMC5853690; doi:10.3390/nano8020057)

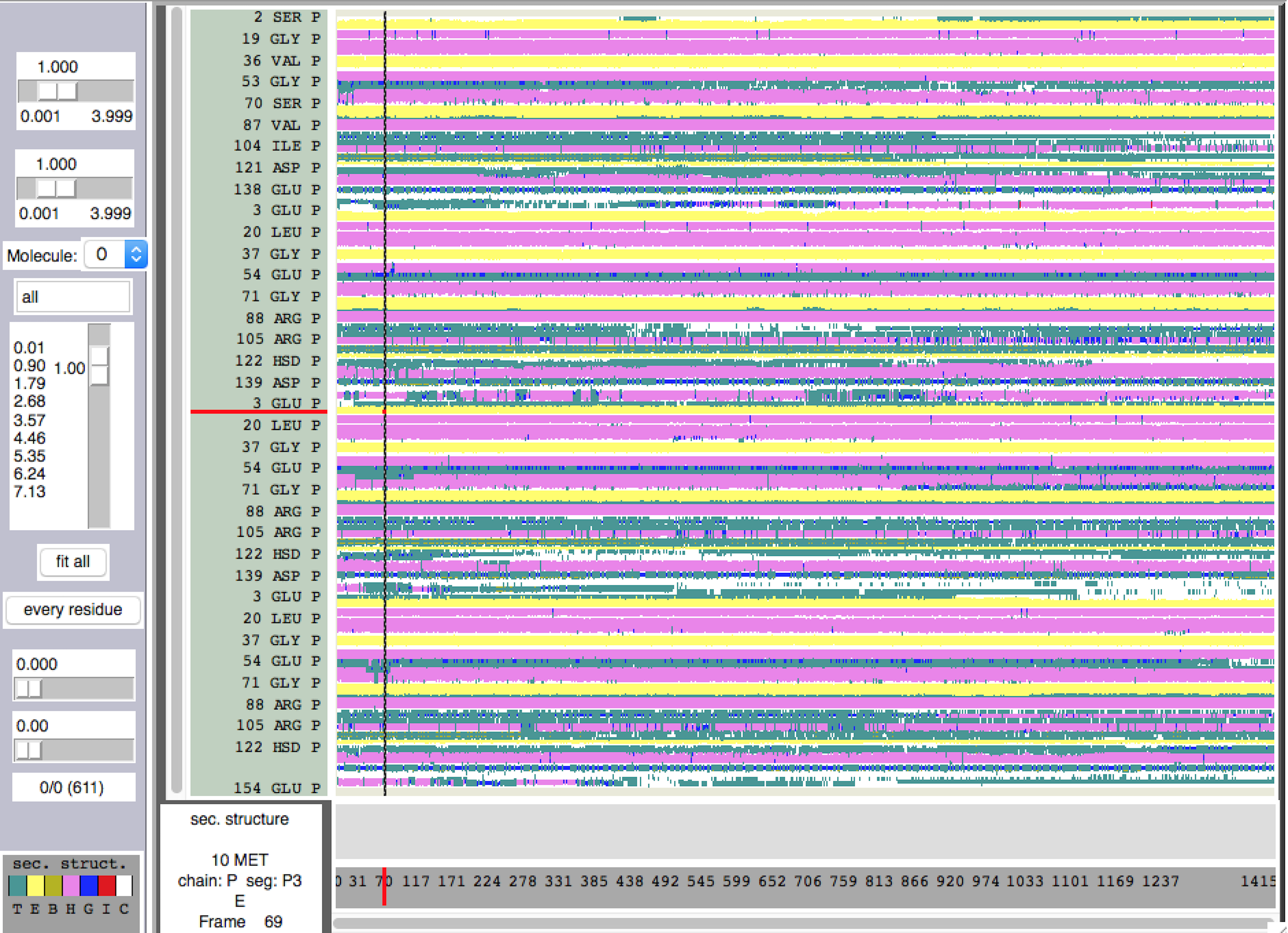

Supplement: Supplementary file 1 [file nanomaterials-08-00057-s001.zip › supplementary/Macwan.Fig_S1.tiff]

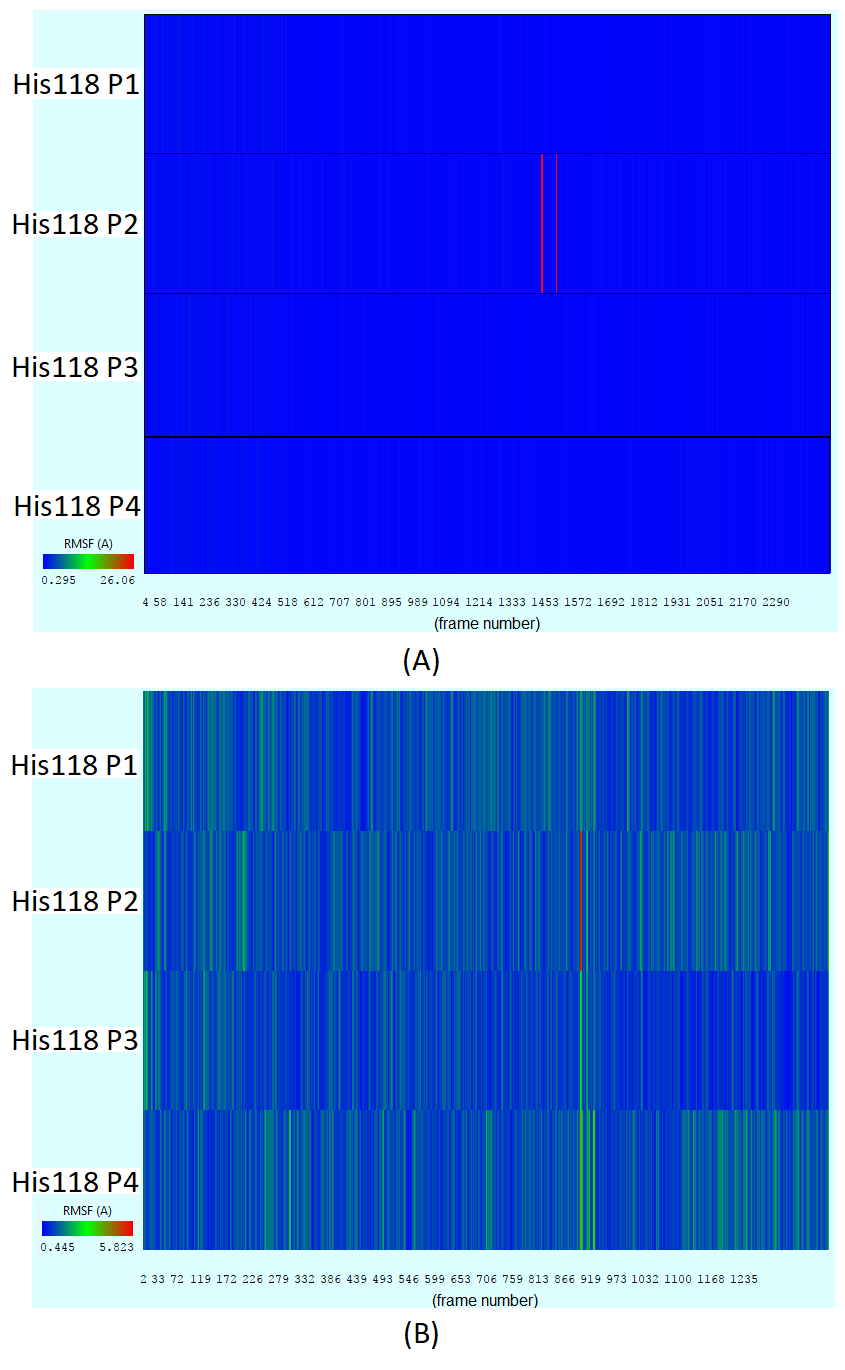

Supplement: Supplementary file 1 [file nanomaterials-08-00057-s001.zip › supplementary/Macwan.Fig_S2_new.tiff]

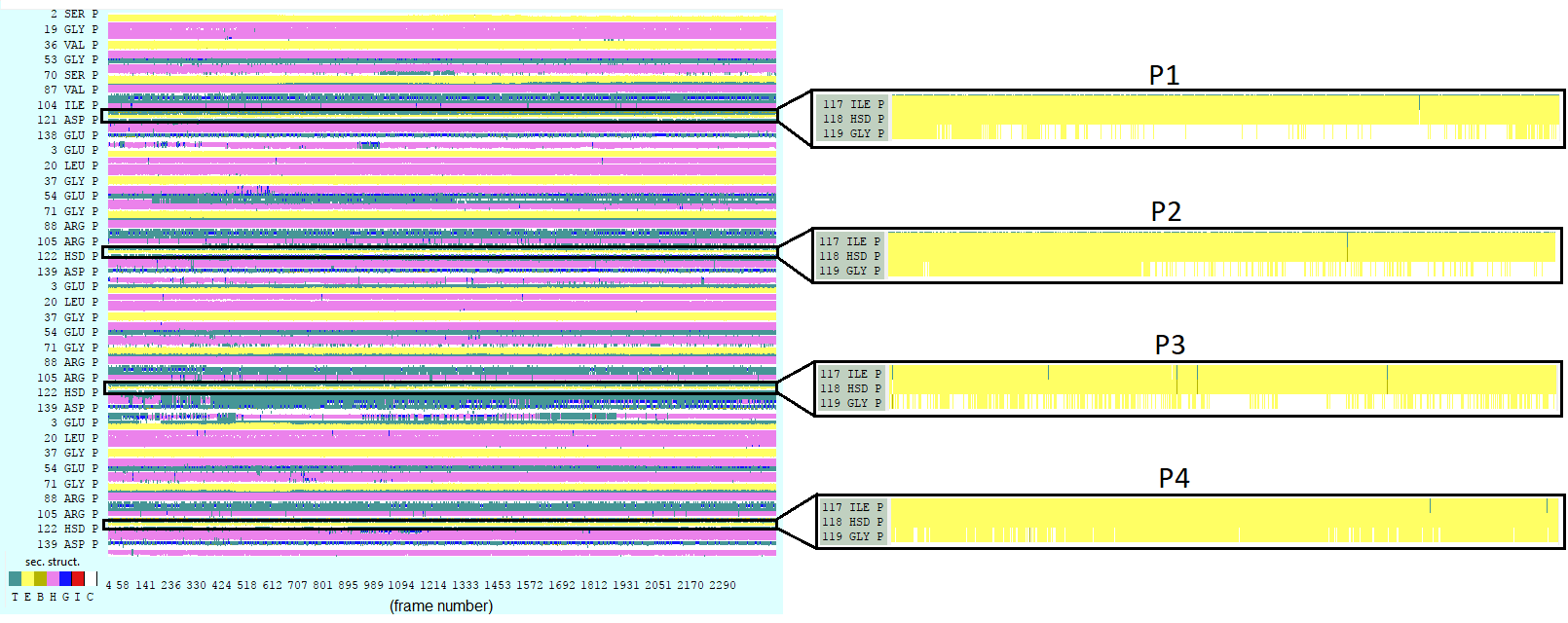

Supplement: Supplementary file 1 [file nanomaterials-08-00057-s001.zip › supplementary/Macwan.Fig_S3_new.tiff]
